# Neural Network-Based Coronary Heart Disease Risk Prediction Using Feature Correlation Analysis

**DOI:** 10.1155/2017/2780501

**Published:** 2017-09-06

**Authors:** Jae Kwon Kim, Sanggil Kang

**Affiliations:** Department of Computer Engineering, Inha University, Incheon, Republic of Korea

## Abstract

**Background:**

Of the machine learning techniques used in predicting coronary heart disease (CHD), neural network (NN) is popularly used to improve performance accuracy.

**Objective:**

Even though NN-based systems provide meaningful results based on clinical experiments, medical experts are not satisfied with their predictive performances because NN is trained in a “black-box” style.

**Method:**

We sought to devise an NN-based prediction of CHD risk using feature correlation analysis (NN-FCA) using two stages. First, the feature selection stage, which makes features acceding to the importance in predicting CHD risk, is ranked, and second, the feature correlation analysis stage, during which one learns about the existence of correlations between feature relations and the data of each NN predictor output, is determined.

**Result:**

Of the 4146 individuals in the Korean dataset evaluated, 3031 had low CHD risk and 1115 had CHD high risk. The area under the receiver operating characteristic (ROC) curve of the proposed model (0.749 ± 0.010) was larger than the Framingham risk score (FRS) (0.393 ± 0.010).

**Conclusions:**

The proposed NN-FCA, which utilizes feature correlation analysis, was found to be better than FRS in terms of CHD risk prediction. Furthermore, the proposed model resulted in a larger ROC curve and more accurate predictions of CHD risk in the Korean population than the FRS.

## 1. Introduction

According to the World Health Organization (WHO), coronary heart disease (CHD) is one of the most dangerous diseases in the world. According to the WHO, around 17.7 million people died from CHD in 2015 [1]. CHD includes hyperlipidemia, myocardial infarction, and angina pectoris [2–4]. In general, medical experts arrive at diagnoses based on electrocardiography, sonography, angiography, and blood test results. CHD is not easily diagnosed during the early disease stage [5–8], but for effective treatment, its early diagnosis is important [9]. However, diagnoses are made based on medical experts' personal experiences and understanding of the disease, which increase the risks of errors, delay appropriate treatment, increase treatment times, and substantially increase costs. In order to solve these problems, many studies have been conducted on clinical decision support systems [10] using various techniques, such as data mining and machine learning [11–15]. Of the machine learning techniques that have been used to predict CHD, neural network (NN) is popularly used to improve performance accuracy [9, 16–20]. NN is good at generalizing data without domain knowledge of CHD prior to training. In addition, by analyzing complex data, NN makes it possible to discover new patterns and information related to CHD [21–23].

Although the NN-based systems mentioned above have provided meaningful results based on clinical experiments, medical experts remain dissatisfied with NN, because of its “black-box” characteristics [24–26], that is, predictors are trained without knowledge of relationships between input features and NN outputs. Many CHD-related features are used to train CHD predictors. Unnecessary or unimportant features for predicting CHD can be included during predictor training. In this case, when the new data is input, it does not predict correctly.

In this paper, we propose an NN-based CHD risk prediction method based on feature correlation analysis (NN-FCA), which includes two processes, that is, feature selection and feature correlation analysis.

- First, during the feature selection stage, we ranked features with respect to their importance for predicting CHD risk. Rankings were calculated using feature sensitivity in a trained NN. Based on these rankings, NN was retrained after eliminating the lowest ranked features in a stepwise manner. This process was continued until the performance of the NN degraded as compared with the previous stage. Once necessary features were obtained using this process, we analyze the NN to know relationship between the features in generating NN output in order to model an NN predictor which can avoid the black-box style training.
- Second, during the feature correlation analysis stage, we analyzed features to identify feature relations and determine whether they were correlated with NN predictor outputs. If features were affected on contribution to predictor output by changing in any of them, features were considered correlated. The NN-based CHD predictor using feature correlation analysis is trained in the way that correlated features are connected in coupled and uncorrelated features are decoupled.

To prove the predictive accuracy of our method, we used the 6th Korea National Health and Nutrition Examination Survey (KNHANES-VI) dataset [27] and evaluated the performances between Framingham risk scores (FRSs) [28, 29], other machine learning techniques, and proposed NN-FCA.

The remainder of this paper consists of the following: Chapter 2 describes the proposed method; Chapter 3 detains results; Chapter 4 provides a discussion; and finally, our conclusions are stated in Chapter 5.

## 2. Method

**Figure 1:**
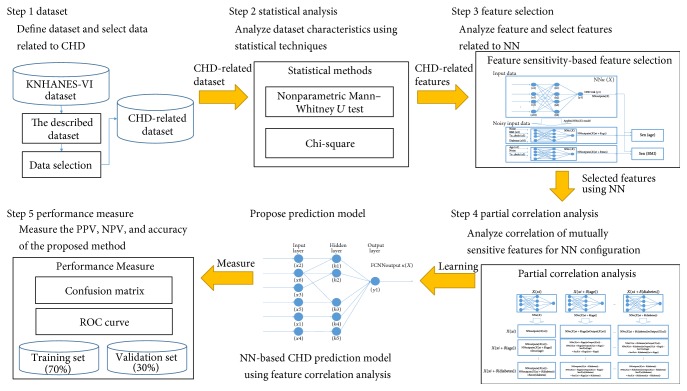
Study design.

The study design is shown in Figure 1. During step 1, KNHANES-VI dataset was examined and data was selected. In step 2, statistical analysis was performed to identify features related to CHD risk. In step 3, predictors of CHD risk were selected using feature sensitivity-based feature selection. In step 4, NN-based CHD risk predictors were trained using feature correlation analysis of features. In step 5, performance measurements were made to validate NN-based CHD risk predictions using feature correlation analysis.

### 2.1. Dataset

The KNHANES-VI was conducted by the Korea Centers for Disease Control and Prevention. KNHANES identifies the health and nutritional status of the population that provides the statistics required to assess whether health policies are being effectively delivered. It also provides statistical data on smoking, drinking, physical activity, obesity, and disease requested by the World Health Organization (WHO) and the Organization for Economic Cooperation and Development (OECD) [27].

We use the KNHANES-VI dataset to perform CHD risk prediction. Input variables for training were age, sex, body mass index (BMI), total cholesterol (To_chole), HDL cholesterol, systolic blood pressure (SBP), diastolic blood pressure (DBP), triglyceride, hemoglobin, thyroid disease (TD), chronic renal failure (CRF), hepatitis type B (H_B), hepatitis type C (H_C), cirrhosis, smoking, and diabetes. The output variables used were CHD risk-related variables, that is, hypertension, dyslipidemia, stroke, myocardial infarction, and angina. When these five diseases are not present and do not exist, CHD is of low risk, but if one of the five is present, CHD is of high risk. 8108 record set of KNHANES-VI was used for the experiment. We excluded 3324 uncertain (nonrespondent, “Null” value) respondents and 638 records of individuals under 30 years old. The final CHD-related dataset comprised 4146 records.

### 2.2. Statistical Analysis

The nonparametric Mann–Whitney *U* test (continuous features) and the chi-square (categorical features) were used to compare age, sex, BMI, To_chole, HDL, SBP, DBP, triglyceride, hemoglobin, TD, CRF, H_B, H_C, cirrhosis, smoking, and diabetes in the low- and high-risk groups. The statistical analysis was performed using IBM SPSS Ver. 22.0 [30]. Several preoperative features were compared to determine the most effective method of CHD risk prediction.

Confusion matrix and receiver operating characteristics (ROC) curve [31] were used for performance comparison. Confusion matrix provides a means of evaluating the performance of the classifier as shown in Table 1 [32]: positive predictive value (PPV), negative predictive value (NPV), and accuracy (1). PPV and NPV are the proportions of positive and negative results with true positive or true negative results, respectively. PPV and NPV describe the performance of diagnostic tests or other statistical measures [33]. The accuracy of a measurement system is the degree of closeness of measurements of a quantity to that quantity's true value [34]. It is constructed for output variable (CHD low risk, CHD high risk) in the validation dataset of each analysis. The limit of significance for all tests is *P* < 0.05.

(1) $$\begin{array}{c} PPV=\frac{TP}{TP+FP}\\ NPV=\frac{TN}{TN+FN}\\ Accuracy=\frac{TP+TN}{TP+FP+FN+TN}. \end{array}$$

### 2.3. Feature Selection

**Figure 2:**
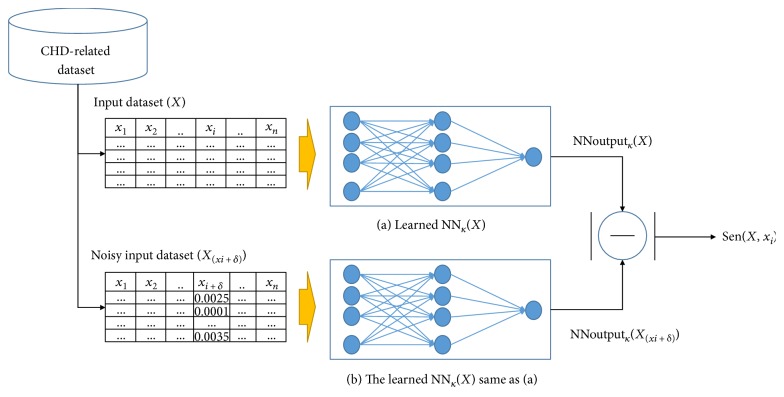
A schematic diagram of calculating the feature sensitivity using NN.

From *n* features extracted for classifying low and high risks, we select features based on importance in contribution to good classification. The importance of each feature is measured by feature sensitivity from a trained NN predictor. The *i*th feature sensitivity, denoted as Sen(*X*, *x_i_*), is calculated by an average of NN output changes between original dataset and noisy dataset which is generated by adding a very small noise (denoted as *δ*) to *x_i_*. The *i*th feature sensitivity is

(2) $$\begin{array}{c} Sen\left(X,x_{i}\right)=\frac{1}{N}\sum_{\forall_{k}}\left|{NNoutput}_{k}\left(X_{\left(x_{i}+\delta \right)}\right)‐NNoutput_{k}\left(X\right)\right|, \end{array}$$

where NNoutput*_k_*(*X*) and NNoutput*_k_*(*X*_(*xi + δ*)_) are the outputs for the input, *k*, with an original input dataset, *X*, and the output with a noisy input (*X*_(*xi + δ*)_) obtained by adding a very small amount of noise *δ* to the *i*th feature, respectively. All feature sensitivities were calculated individually with one feature sensitivity. The *δ* value was generated randomly within the range [a1, 0.0010].  Figure 2 presents a schematic diagram of the methodology for calculating the feature sensitivity using NN. All feature sensitivities were sorted in a descending order, and the feature with the lowest sensitivity of the feature set was eliminated. The NN was retrained using the remaining features and then verified to determine whether the performance is not degraded compared to that of the original NN trained using all features. If the performance is not degraded, then the aforementioned process repeats until the necessary features are determined.

### 2.4. Feature Correlation Analysis

**Figure 3:**
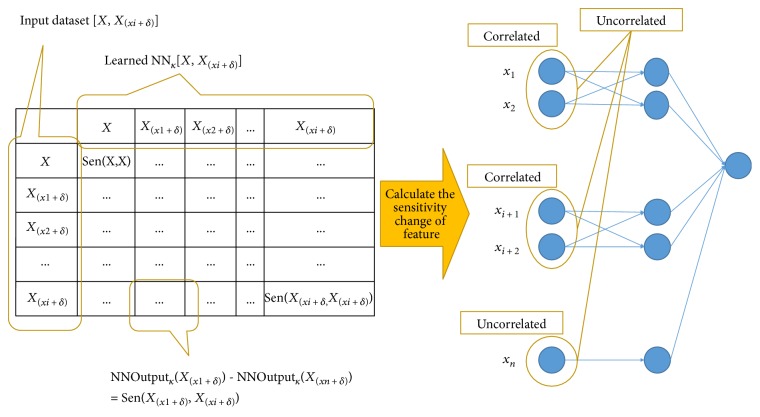
An example of NN predictor using the feature correlation analysis.

To overcome the performance limitation of NN due to the characteristics of black-box training [24–26], prior information on the correlation relationship among the features was acquired using the feature sensitivity change in generating NNoutputs. The correlated features are connected to the hidden layer in a coupled connection. On the other hand, the uncorrelated features are connected in uncoupled connection. The sensitivity of a feature in a trained *NN* means the relative importance index in generating NNoutput. This contains the intention that if the magnitude of a feature increases, the importance of the feature increases while training NN. Moreover, if the magnitude of the increase in the feature affects the other features significantly, the corresponding features can be considered to be correlated with each other. To determine if the features are correlated or uncorrelated, this study examined the changes in feature sensitivity, as seen in the algorithm in Pseudocode 1.  Figure 3 gives an example of the NN prediction model trained based on the feature relations, such as correlated and uncorrelated.

## 3. Result

### 3.1. Characteristics

Table 2 lists the distribution of the preoperative parameters between the people at low risk and high risk of CHD.

The median age of the 4146 subjects was 52 years (range: 30–92; mean: 52.501). The median low-risk age and high-risk age were 47 years (range: 30–87; mean: 48.60) and 64 years (range: 30–92; mean: 63.11), respectively. The median BMI was 23.68 (range: 15.302–41.304; mean: 23.969). The median low-risk BMI and high-risk BMI were 23 (range: 15–40; mean: 23.594) and 25 (range: 16–41; mean: 25.004), respectively. The median To_chole level was 189 mg (range: 79–525; mean: 190.974). The median low-risk To_chole level and high-risk To_chole level were 190 mg (range: 89–384; mean: 191.738) and 185 mg (range: 79–525; mean: 188.898), respectively. The median HDL was 50 mg (range: 22–118; mean: 51.843). The median low-risk HDL and high-risk HDL were 51 mg (range: 22–111; mean: 52.642) and 48 mg (range: 23–118; mean: 49.671), respectively. The median SBP level was 117 mmHg (range: 75–219; mean: 118.979). The median low-risk SBP level and high-risk SBP level were 113 mmHg (range: 75–209; mean: 155.583) and 127 mmHg (range: 88–219; mean: 128.209), respectively. The median DBP was 75 mmHg (range: 10–137; mean: 75.822). The median low-risk DBP level and high-risk DBP level were 75 mmHg (range: 44–137; mean: 75.61) and 76 mmHg (range: 10–118; mean: 76.397), respectively. The median triglyceride level was 112.5 mmol/L (range: 20–1868; mean: 139.236). The median low-risk triglyceride level and high-risk triglyceride level were 106 mmol/L (range: 20–1868; mean: 131.570) and 129 mmol/L (range: 28–1397; mean: 160.0744), respectively. The median hemoglobin level was 13.9 mg/dl (range: 6.7–19.1; mean: 13.981). The median low-risk hemoglobin level and high-risk hemoglobin level were 14 mg/dl (range: 7–19; mean: 14.057) and 14 mg/dl (range: 7–18; mean: 13.989), respectively. The difference between the 2 groups (low risk and high risk) in age, BMI, To_chole, HDL, SBP, DBP, and triglyceride was significant (independent *t*-test): *P* = 0.001 (age), *P* = 0.001 (BMI), *P* = 0.024 (To_chole), *P* = 0.001 (HDL), *P* = 0.001 (SBP), *P* = 0.035 (DBP), *P* = 0.001 (triglyceride), and *P* = 0.206 (hemoglobin). The 4146 subjects were classified according to sex as female (1777) and male (2369). The TD was classified as no (4073) and yes (73). The CRF was classified as no (4134) and yes (12). The H_B was classified as no (4117) and yes (29). The H_C was classified as no (4143) and yes (3). Cirrhosis was classified as no (4136) and yes (10). Smoking was classified as no (3322) and yes (824). Diabetes was classified as no (2625). An impaired fasting glucose was classified as no (994) and yes (527). The difference between the 2 groups (low risk and high risk) in sex, TD, CRF, H_B, H_C, cirrhosis, smoking, and diabetes triglyceride was significant (chi-square test): *P* = 0.893 (sex), *P* = 0.370 (TD), *P* = 0.022 (CRF), *P* = 0.933 (H_B), *P* = 0.801 (H_C), *P* = 0.349 (cirrhosis), *P* = 0.001 (smoking), and *P* = 0.001 (diabetes).

### 3.2. Feature Sensitivity-Based Feature Selection Result

**Figure 4:**
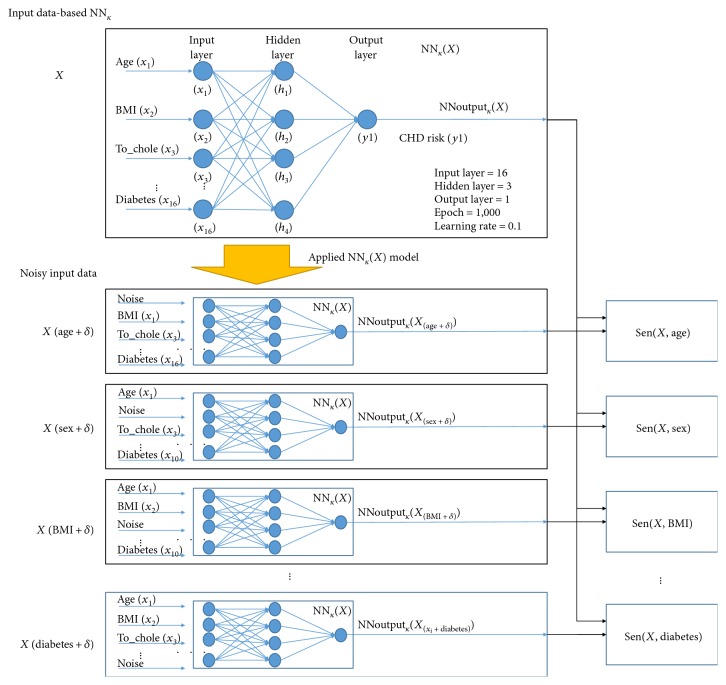
Calculation process of the feature sensitivity.

NN*_k_*(*X*) consisted of 16 input nodes, 4 hidden nodes, and one output node. Noisy data (*x_i_-δ*) were applied to the trained NN*_k_*(*X*) to calculate the sensitivity of each feature.  Figure 4 outlines the calculation process of the feature sensitivity.

Table 3 presents the results of the feature sensitivity. From the Table, To_chole (0.100), age (0.081), SBP (0.073), and DBP (0.049) are considered the important features for CHD risk predictor. The NN is retrained by removing the lowest ranked feature one at a time until the performance of the NN degrades, as shown in Table 4. The best performance was obtained when only seven features (sex, hemoglobin, TD, CRF, H_B, H_C, and cirrhosis) were removed, with an 81.163% accuracy of predicting CHD.

### 3.3. NN-Based CHD Risk Predictor Using Feature Correlation Analysis

**Figure 5:**
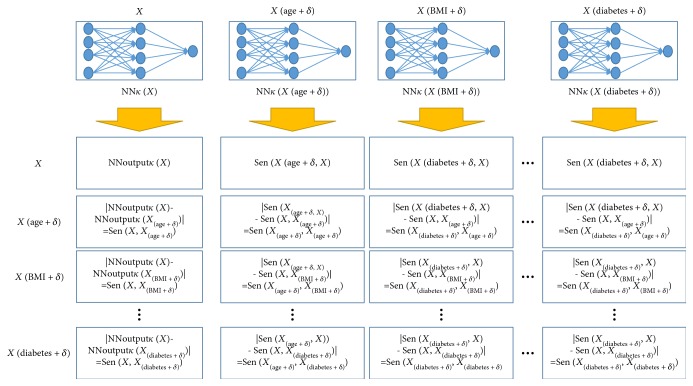
The process of feature correlation analysis.

**Figure 6:**
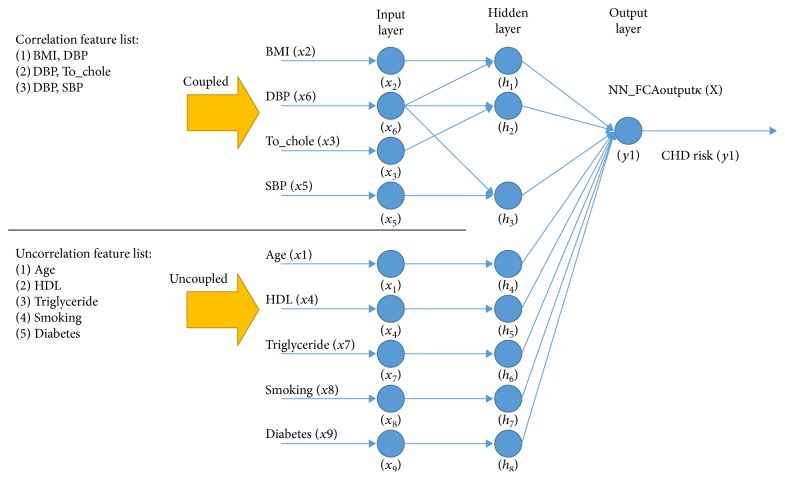
NN-based CHD prediction using feature correlation analysis.

From the result in Section 3.2, the nine features (age, BMI, To_chole, HDL, SBP, DBP, triglyceride, smoking, and diabetes) were selected and used for feature correlation analysis, as shown in Figure 5. The correlated features of each feature were determined according to the mutual effects on the sensitivity changes. In other words, the correlated features influenced their sensitivity changes in one another due to the amplification of a single feature. For example, the change in feature sensitivity of SBP was 0.017 when it was amplified, which is denoted as *X*(_SBP*+δ*_), as listed in Table 5. The amplification on SBP is believed to have been affected by the sensitivity changes of three features, such as BMI (0.025), To_chole (0.042), and DBP (0.017), because they showed much or higher sensitivity changes than the average sensitivity change (0.017) of all the features. To verify the mutuality of the correlation, the sensitivity change of SBP was analyzed according to the amplification on BMI, To_chole, and DBP, respectively. For the amplification on BMI (*X*(_SBP*+δ*_)), the sensitivity change of SBP is 0.007, which is much less than the average sensitivity change (0.012) of all features. Therefore, BMI is not considered to be correlated with SBP. For the amplification on To_chole (*X*(_To_chole*+δ*_)), SBP was not correlated, similar to the BMI. On the other hand, for the amplification of DBP (*X*(_DBP*+δ*_)), the sensitivity change of SBP was 0.035, which is larger than the average sensitivity change (0.022) of all features. Overall, the analysis showed that the SBP and DBP are correlated with each other. The correlated features for the remaining features were examined in the same way. Based on the correlation of features, the NN-based CHD risk predictor, in which the correlated features are coupled in connection to the hidden layer, was modelled, as seen in Figure 6. For example, BMI and DBP were coupled in connection to the hidden layer because both are correlated with each other.

### 3.4. Performance Measure

The performance of the proposed NN-based CHD risk prediction was examined using feature correlation analysis, and the results were compared with those obtained by feature correlation analysis (NN_FCA) with logistic regression (LR), neural network (NN), and Framingham risk score (FRS) [28], using the performance metrics, such as confusion matrix (positive predictive value (PPV), negative predictive value (NPV), and accuracy) and ROC curve. The experimental dataset was divided into training set (70%) and validation set (30%).  Table 6 lists the results of the performance measure.

From Table 5, FRS showed a lower performance with an accuracy of 28.87%. LR and NN gave high performance (80.32% and 81.09%, resp.), but the performance was lower than that of NN_FCA. NN_FCA showed the best performance compared to the other models in both the training set and validation set (87.63% and 82.51%). The PPV and NPV also showed the highest NN_FCA (71.29% and 85.70%, resp.) than the other models. The accuracy of NN_FCA was highest at 82.51% because the correlation relationship of the features is trained while training NN_FCA.

**Figure 7:**
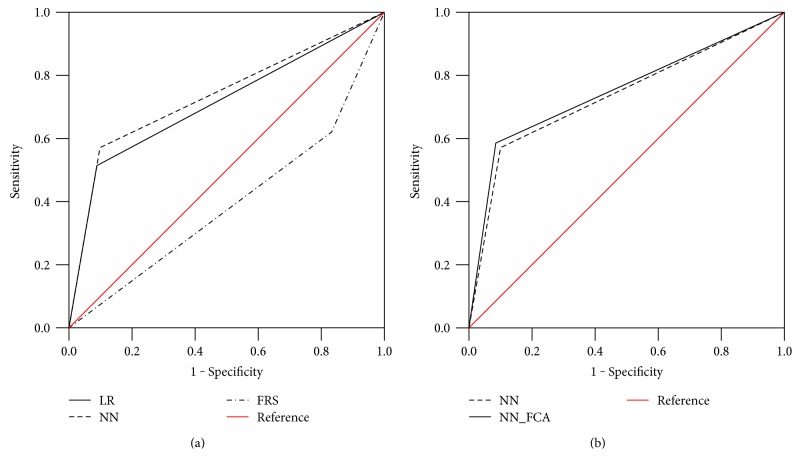
Result of ROC curve (a) compared to LR, NN, and FRS; (b) compared to NN and NN_FCA.

The results of the ROC curve are shown in Table 7 and Figure 7. As shown on the left of the figure, FRS has a very low ROC area of 0.393 ± 0.010. Because FRS is a statistical method suitable for a specific population and environment, it appears to be unfit for the Korean population. LR and NN were 0.713 ± 0.010 and 0.735 ± 0.010, respectively. Here, NN was found to be effective for predicting the CHD risk, as reported in a previous study [17, 35]. On the other hand, as shown on the right of the figure, NN_FCA was 0.749 ± 0.010, which was better than the existing NN, because it removes the unnecessary features when training the prediction model. In other words, the sensitivity-based feature selection can effectively detect the features associated with a prediction of the risk of CHD.

As a result, the error rate can be reduced using NN_FCA because it removes the unnecessary connections between the nodes in NN. Therefore, NN_FCA is excellent in terms of the performance accuracy. The proposed NN_FCA is effective for predicting the risk of CHD.

## 4. Discussion

NN is a training method that imitates the human brain and is a very successful technique for predicting the relationship between the input values and target values. In addition, it is a predictive model for supporting a back propagation method and a powerful technique that can help in determining the support involved in the problems of classification, inference, prediction, and sequential reasoning [36, 37]. Substantial research has attempted to predict the CHD risk; LR and NN are used typically in machine learning. The prediction performance degrades because unnecessary features are considered during training LR and NN [9, 16–20]. The proposed method solves this problem by removing the unnecessary features using sensitivity-based feature selection.

The most popular decision support of the risk of CHD is the Framingham risk score (FRS) [28], which provides the CHD risk index with a statistical technique using the patients' demographics and various medical examination information. Currently, the accuracy of the FRS is 28.87%, as evaluated using the KNHANES-VI dataset [27]. The FRS has difficulty in reflecting the environments, which change with time, and is limited to patients in a specific region because it uses the U.S. patients' data collected from 1960 to 1970 [29].

Many studies have been conducted to predict the risk of CHD using machine learning. Arabasadi et al. [35] proposed a hybrid neural network genetic for a CHD risk prediction in 2017. In this work, the input features were selected using a genetic algorithm and the CHD predictor was then modelled with a neural network. Narain et al. [9] developed a CHD risk prediction system modelled with the quantum neural network in 2016. This work increased the quantum interval according to the error value of the output layer during training and provided weights to the sigmoid function. Verma et al. [16] proposed a novel hybrid method, in which feature selection, particle swarm optimization, and K-means were used for a CHD prediction in 2016. They finally employed supervised learning, such as NN, LR, and fuzzy unordered rule induction as well as a C4.5 decision tree for classification. Zhao and Ma [17] proposed an intelligent noninvasive diagnosis system based on empirical mode decomposition-Teager energy operator to estimate the instantaneous frequency of diastolic murmurs and back propagation NN to classify the murmurs in 2008. They worked on classifying a normal group and CHD group according to the electrocardiogram (ECG) signal for diastolic murmurs. Akay [18] proposed a CHD predictor modelled using a NN in 1992. They presented a clinical demonstration from the data of 100 patients. Kukar et al. [19] proposed a CHD prediction system using the ECG data and modelled it with a Bayesian NN. Detrano et al. [20] developed a CHD prediction system modelled from the data of 425 patients using the LR technique. As mentioned above, CHD prediction studies using NNs are ongoing.

This study was conducted to predict the risk of CHD in Koreans. In general, heart disease is influenced by age, sex, BMI, total cholesterol, HDL, systolic blood pressure, diastolic blood pressure, smoking, and diabetes [38–46]. In Koreans, CHD was not found to be associated with sex, hemoglobin, thyroid disease, H_B, H_C, or cirrhosis disease (*P* value < 0.05). On the other hand, triglyceride and CRF were associated with CHD (*P* value = 0.035). Triglyceride is an important factor in predicting the risk of CHD. This study confirmed that triglyceride is a very important factor for CHD in Koreans. In addition, the results of NN-based CHD risk prediction using feature correlation analysis showed that SBP and DBP are correlated. This is reasonable because both have similar characteristics. In addition, BMI and DBP are closely related, that is, obese people have high blood pressure in general [47]. In addition, the relationship between DBP and total cholesterol affects CHD [48]. The proposed NN-based CHD risk prediction using feature correlation analysis showed higher accuracy (82.51%) in a CHD prediction compared to the other models and proved to be more useful than the FRS applied in the past.

## 5. Conclusion

This paper proposed an NN-based CHD risk prediction using feature correlation analysis (NN-FCA) and experimented with the KNHANES-VI dataset. The proposed model will improve the CHD risk and decision support for suitable treatment. Sex, hemoglobin, thyroid disease, H_B, H_C, and cirrhosis were not associated, whereas triglyceride and CRF were closely related to CHD. In addition, triglyceride is a very important factor in the risk of CHD in Koreans. Furthermore, the correlated features are BMI and DBP, DBP and total cholesterol, and SBP and DBP. The proposed model was as good as FRS in terms of the CHD risk prediction. Compared to the validation of the FRS for the Korean population, the proposed model resulted in a larger ROC curve and more accurate CHD risk prediction.

The proposed model acknowledging such characteristics was developed, which may aid in the prevention of heart disease in these individuals. This might deliver great benefit to people in terms of predicting, beyond a simple prediction of the CHD risk and the quantitative survival time. Furthermore, a self-diagnosis algorithm or a similar clinical decision support system could be developed and applied meaningfully if the NN-FCA can be applied to diseases other than CHD.

## Figures and Tables

**Pseudocode 1:**
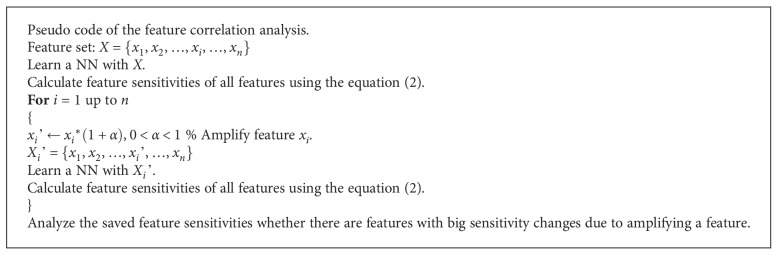


**Table 1:** Confusion matrix.

Confusion matrix		Prediction	
		Positive	Negative
Actual	Positive	True positive (TP)	False positive (FP)
	Negative	False negative (FN)	True negative (TN)

**Table 2:** Characteristics (continuous variable: mean; categorical variable: count).

Feature	Low risk (3031 people)	High risk (1115 people)	*P* value
Age	48.600	63.110	0.326
Sex			0.893
Male	1301	476	
Female	1739	639	
BMI	23.594	25.004	0.001
To_chole	191.738	188.898	0.024
HDL	52.642	49.671	0.001
SBP	115.583	128.210	0.001
DBP	75.610	76.397	0.035
Triglyceride	131.570	160.074	0.001
Hemoglobin	14.057	13.989	0.206
TD			0.370
No	2981	1092	
Yes	50	23	
CRF			0.002
No	3027	1092	
Yes	4	23	
H_B			0.933
No	3010	1107	
Yes	21	8	
H_C			0.801
No	3029	1114	
Yes	2	1	
Cirrhosis			0.349
No	3025	1111	
Yes	6	4	
Smoking			0.001
No	2350	972	
Yes	681	143	
Diabetes			0.001
No	2167	458	
Impaired fasting glucose	671	323	
Diabetes	193	334	

**Table 3:** Results of feature sensitivity analysis.

Features	Sensitivity	NNoutput-NNoutput(*xi* + *δ*)	Rank
NN*_k_*(*X*)	0.815		
NN*_k_*(*X*_(*xi−*age)_)	0.734	0.081	2
NN*_k_*(*X*_(*xi−*sex)_)	0.726	0.008	11
NN*_k_*(*X*_(*xi−*BMI)_)	0.769	0.038	5
NN*_k_*(*X*_(*xi−*To_chole)_)	0.677	0.100	1
NN*_k_*(*X*_(*xi−*HDL)_)	0.703	0.013	8
NN*_k_*(*X*_(*xi−*SBP)_)	0.729	0.073	3
NN*_k_*(*X*_(*xi−*DBP)_)	0.693	0.049	4
NN*_k_*(*X*_(*xi−*triglyceride)_)	0.753	0.013	7
NN*_k_*(*X*_(*xi−*hemoglobin)_)	0.796	0.006	12
NN*_k_*(*X*_(*xi−*TD)_)	0.806	0.003	13
NN*_k_*(*X*_(*xi−*CRF)_)	0.802	0.010	10
NN*_k_*(*X*_(*xi−*H_B)_)	0.813	0.001	15
NN*_k_*(*X*_(*xi−*H_C)_)	0.813	0.001	16
NN*_k_*(*X*_(*xi−*cirrhosis)_)	0.812	0.002	14
NN*_k_*(*X*_(*xi−*smoking)_)	0.802	0.012	9
NN*_k_*(*X*_(*xi−*diabetes)_)	0.786	0.024	6

**Table 4:** Results of NNs eliminating the lowest ranked features (%).

Without features	Accuracy
Without 1 (H_C) feature	77.743
Without 2 (H_C and H_B) features	78.518
Without 3 (without 2 features and cirrhosis) features	80.644
Without 4 (without 3 features and TD) features	80.920
Without 5 (without 4 features and hemoglobin) features	81.120
Without 6 (without 5 features and sex) features	81.141
Without 7 (without 6 features and CRF) feature	81.163
Without 8 (without 7 features and smoking) features	81.018
Without 9 (without 8 features and HDL) features	80.921
Without 10 (without 9 features and triglyceride) features	80.222
Without 11 (without 10 features and diabetes) features	79.522
Without 12 (without 11 features and BMI) features	79.209

**Table 5:** Nine features (age, BMI, To_chole, HDL, SBP, DBP, triglyceride, smoking, and diabetes) are selected and used for feature correlation analysis.

Input dataset	Learned NN*_k_*								
	*X*_(age*+δ*)_	*X*_(BMI*+δ*)_	*X*_(To_chole*+δ*)_	*X*_(HDL*+δ*)_	*X*_(SBP*+δ*)_	*X*_(DBP*+δ*)_	*X*_(triglyceride*+δ*)_	*X*_(smoking*+δ*)_	*X*_(diabetes*+δ*)_
Age	0.080	0.009	0.016	0.004	0.011	0.008	0.009	**0.022**	0.019
BMI	0.031	0.038	0.019	**0.013**	**0.025**	**0.026**	0.037	0.010	**0.036**
To_chole	0.021	**0.012**	0.094	**0.017**	**0.042**	**0.070**	**0.013**	0.013	**0.064**
HDL	0.011	0.010	0.011	0.011	0.010	0.008	0.009	0.009	0.002
SBP	0.012	0.007	0.001	**0.020**	0.041	**0.035**	0.008	0.013	0.016
DBP	**0.496**	**0.013**	**0.043**	**0.017**	**0.017**	0.021	0.001	**0.029**	**0.045**
Triglyceride	0.009	0.005	0.008	0.008	0.003	0.005	0.009	0.005	0.006
Smoking	0.005	0.004	0.004	0.003	0.003	0.008	0.002	0.012	0.007
Diabetes	0.002	0.006	0.003	0.007	0.008	0.019	0.005	0.009	0.019

Average	0.074	0.012	0.022	0.011	0.017	0.022	0.010	0.014	0.024

Candidates of correlated feature	DBP	To_chole, DBP	DBP	BMI, To_chole, SBP, DBP	BMI, To_chole, DBP	BMI, To_chole, SBP	To_chole	Age, DBP	BMI, To_chole, DBP

**Table 6:** Results of performance measure with training set (%).

	Training set			Validation set		
	PPV	NPV	Accuracy	PPV	NPV	Accuracy
LR	57.24	87.63	86.11	67.53	83.63	80.32
NN	63.04	88.67	87.04	67.55	85.08	81.09
FRS	2.54	85.48	6.67	21.49	54.41	28.87
NN_FCA	67.57	89.00	87.63	71.29	85.70	82.51

**Table 7:** Results of ROC curve using validation set.

			95% Confidence Interval	
	ROC curve	*P* value	Lower bound	Upper bound
LR	0.713 ± 0.010	0.001	0.693	0.732
NN	0.735 ± 0.010	0.001	0.716	0.754
FSNN	0.741 ± 0.010	0.001	0.722	0.760
FRS	0.393 ± 0.010	0.001	0.373	0.414
NN_FCA	0.749 ± 0.010	0.001	0.731	0.768
